# Antiherding in Financial Decision Increases Valuation of Return on Investment: An Event-Related Potential Study

**DOI:** 10.1155/2017/4760930

**Published:** 2017-05-28

**Authors:** Cuicui Wang, Jia Jin, João Paulo Vieito, Qingguo Ma

**Affiliations:** ^1^School of Management, Hefei University of Technology, Hefei, China; ^2^Neuromanagement Lab, Zhejiang University, Hangzhou, China; ^3^School of Management, Zhejiang University, Hangzhou, China; ^4^Business School, Ningbo University, Ningbo, China; ^5^Academy of Neuroeconomics and Neuromanagement at Ningbo University, Ningbo, China; ^6^School of Business Studies, Polytechnic Institute of Viana do Castelo, Viana do Castelo, Portugal

## Abstract

Using event-related potentials, this study investigated how financial herding or antiherding affected the valuation of subsequent outcomes. For each trial, subjects decided whether to buy the stock according to its net money flow information which could be used to reflect the strength of buying power or selling power of the stock. The return on investment (ROI) as feedback included the increase or decrease percentage after subjects' responses. Results showed that, compared with herding, antiherding induced larger discrepancies of FRN and P300 amplitude between positive ROI and negative ROI, indicating that individuals under antiherding condition had stronger motivation and paid more attention in the evaluation process of ROI. Moreover, only for positive ROI, the amplitudes of FRN and P300 were modulated by two kinds of behaviors. We suggested that individuals making antiherd decisions were more confident with their own ability and choices, which reduced the positive outcome prediction error and gave more mental resources to evaluate positive outcome. However, negative outcomes evoked no different motivational meaning and negative emotion for individuals between herding and antiherding. The study may provide new insights into neurocognitive processes of herding and antiherding in financial market.

## 1. Introduction

Herding refers to this phenomenon, in which individuals are strongly influenced by the decisions of others and do what others are doing [1]. In financial investment, the first reason why individual investors “follow the herd” is that other investors may know something about the return on the investment (ROI) and their actions reveal this information. The second reason is that individuals may have an intrinsic preference for herding [2]. There is also quite a lot of antiherding behaviour which describes the social and economic situations in which individuals excessively contradict public information [3]. The decision makers of antiherding go against the herd and ignore or contradict advice even against their own belief. Antiherding is attractive if there is at least some probability that individuals cannot recognize who was right and who was wrong after they have made decisions in the first period. Effinger and Polborn (2001) demonstrated decision makers always oppose their predecessor's report if the value of antiherding strategy is sufficiently large [4]. In financial investment, according to the decisions other investors make, we cannot recognize whether they are right or not. So the herding and antiherding exist all at once in financial decision-making.

Most research of herding and antiherding has been done in behavioral and modeling studies [2, 5–7]. However, recent neuroimaging and electrophysiological studies also have investigated their neural basis [8, 9]. Berns and his colleagues used functional magnetic resonance imaging (fMRI) technique to investigate social herding and found that herding was associated with functional changes in an occipital-parietal network [9]. Klucharev et al. (2009) found that the herding (conformity) was involved in the activity of the posterior medial frontal cortex and ventral striatum areas which were known to be associated with reward-monitoring neural circuitry [10]. Using event-related potentials (ERPs), Chen et al. studied the neural substrates of conflicts in antiherd choices in purchasing books online. The research showed that a strong negative deflection of ERP in the time window 300–600 ms after the stimulus onset was recorded when participants made antiherd choices [11]. In a novel group gambling task, when the participants' choices were different from others, the feedback-related negativity (FRN) (one of ERP components) was enhanced, which demonstrated that being independent for individuals was aversive [12]. However, most previous research on the neural basis of herding was not associated with financial conformity, but associated with social conformity. And they focused on different level of conflict evoked by changing the number of group members making different choices from the participant's choice [8, 13] but did not focus on other forms of herding. In addition, few studies paid attention to how herding or antiherding behavior impacted on subsequent outcome evaluation. To date in financial investment, few studies have explored the temporal dynamics of how herd behavior shapes investors' valuation of return on investment (ROI), especially compared with antiherd behavior, which is the purpose of this paper.

To investigate how herding and antiherding modulated individual's evaluation of ROI, we adopted ERPs and a simplified investment task to explore this cognitive process. In financial decision-making, individual's choice was not only affected by the group opinions, but also affected by the capital amount invested by other investors. The index of net money flow information of stocks was used to reflect the behaviors of other investors in prior period, which produced herding or antiherding, because it contained the judgment on the price trend and amount invested which were both important for participants' decision. Money flow, whether money is flowing into (positive money flow) or out (negative money flow) of a particular stock, is an index measuring the strength of money going in and out of a stock in a period [14]. Net money flow equals positive money flow minus negative money flow, which can reflect the general judgments of investors on the price trend and the money she/he invests in the previous stage. There are two categories of net money flow: positive and negative. Positive net money flow means the power of buying is larger, and the behavior to buy this stock with positive net money flow is considered as herding with the behavior not to buy the stock as antiherding, while negative money flow information means the power of selling is larger, and the behavior to buy this stock with negative net money flow information is considered as antiherding with the behavior not to buy the stock as herding. After the individuals' herding or antiherding behavior, the outcomes (gain or loss as ROI) would be evaluated. The ERP method can be employed as a measurement to subjectively evaluate the value of ROI under financial investment background and to explore the temporal dynamics of how antiherd or herding behavior shapes investors' valuation of ROI. Furthermore, the study may provide further explanations for the reason why individual investors “follow the herd” or go against the herd.

In ERPs studies, ERP components which are concerned with outcome evaluation are feedback-related negativity (FRN) and P300. FRN is a negative ERP component occurring at 200–300 ms after the presentation of outcome, which is maximal over medial frontal scalp. The amplitude of FRN is more negative following negative feedback, such as game failure or monetary loss [15–17]. According to the classic reinforcement learning theory, the FRN component represents the transmission of a neural reward prediction error signal from midbrain dopamine neurons to the anterior cingulate cortex (ACC) [18]. It could explain why the unexpected negative outcomes (losses) would elicit a relatively larger FRN as compared to positive outcomes (gains). Other researchers suggest that FRN effect reflects the processes of assessing the motivational/affective impact of outcomes. That is, it reflects the processes of putting subjective values into the outcomes [15, 17, 19]. Previous research demonstrates that attitude, effort, and responsibility of individuals moderate the motivation in the process of evaluating outcomes, which can be reflected by FRN component [20–22]. Larger FRN amplitudes indicate stronger motivational or emotional impact of the current outcome event. Ma et al. (2013) found that high effort in calculation tasks induced larger differentiated FRN responses to the reward and nonreward discrepancy, which suggested that effort might increase subjective evaluation towards subsequent outcome [22]. In this study, compared with herding, antiherding which was against the herd and might make more stresses could augment the motivational significance of the accompanying ROI and then enlarge the amplitude of FRN discrepancy.

The P300, another ERP component, is the most positive deflection in the period of 300–600 ms after onset of feedback, which typically has its maximum magnitude at parietal sites [18, 23, 24]. In many cases, P300 component fits in with the context-updating hypothesis proposed by Donchin and Coles (1981), which suggests that P300 reflects the allocation amount of attention and other cognitive resources [25]. The attention paid to the stimulus or event can modulate the effects of subjective motivational significance and probability on the amplitude of P300 [26, 27]. For the outcome evaluation, P300 played a salient role in this process. Cui et al. (2013) suggested that P300 reflected a later and top-down controlled outcome evaluation process in which factors related to the allocation of attention played a role, while FRN component reflected the early appraisal of outcome on a binary classification basis of good or bad outcomes [28]. The effect of outcome valence on the P300 amplitude does not reach a consensus. Early studies suggested that negative outcome elicited larger P300 amplitude than positive outcomes [29]. However, Yeung and Sanfey (2004) used monetary rewards and found that P300 was sensitive to outcome magnitude but insensitive to outcome valence [24]. In the subsequent study, emotionally valent outcomes are associated with larger P300 than emotionally neutral outcomes, and Hajcak et al. (2005) observed that the positive outcomes evoked larger P300 amplitudes than the negative outcomes in monetary gambling tasks [23]. The effects of motivational significance on P300 amplitude are modulated by the amount of attention paid to the stimulus, which suggested that P300 could encode the motivational/affective significance of the stimuli in the process of outcome evaluation [26]. Researcher also found that the modulated P300 amplitudes evoked during outcome evaluation tasks probably reflected the evaluation of the functional significance of the feedback [30]. We speculated antiherding could affect the allocation of attentional resources in the process of cognitive and affective evaluation of ROI, which could modulate the amplitudes of P300 in feedback stage. With this experiment, it would provide some new insights into the relations between P300 and outcome valence.

In the present study, we used ERPs to investigate the difference between herding and antiherding in valuation of ROI information under financial investment background. The tasks in this experiment were revised and simplified from real investment decision-making. Each participant was asked to choose to buy or not according to the information of net money flow of stocks. Positive net money flow means the power of buying in financial market is larger in a period of time, and negative net money flow means the power of selling in financial market is larger. If she/he chose to buy in positive net money flow or not to buy in negative net money flow, we could classify them as herding. If they chose not to buy in positive net money flow or to buy in negative net money, they could be called antiherding. After participants' responses, the positive or negative ROI was given. The electroencephalogram (EEG) signals were recorded throughout the experiment. Given that FRN reflects motivational/affective evaluation and P300 reflects the allocation of attentional resources which modulates motivational salience, we postulated that the positive/negative FRN and P300 discrepancy of antiherding would be more pronounced than that of herding when observing the ROI.

## 2. Material and Methods

### 2.1. Participants

Twenty-one major undergraduates of Zhejiang University (nine females, all right-handed), aged from 20 to 25 years (M = 22.95 years, s.d. = 1.50 years), participated in this experiment as paid volunteers. They had some knowledge on stock investment but no experience of real investment. All subjects were native Chinese speakers, had normal or corrected to normal vision, and had no history of neurological or psychiatric abnormalities. The research was approved by the Internal Review Board of Zhejiang University Neuromanagement Lab. Before experiment, informed consent was obtained from all volunteers. Behavioral data of first two subjects were not recorded, as some problems were found in the software codes.

### 2.2. Experiment Stimuli and Procedure

Participants sat in a comfortable chair in an electrically shielded and sound-attenuated cabin. A computer display was located 1 m away from their eyes. There was a keypad provided for them to make choices. The experiment consisted of 4 blocks, each containing 100 trials. During the experiment, 200 trials were presented with positive net money flow, half of which had positive ROI (the increase of stock in percentage terms), and the other half of which had negative ROI (the decrease of stock in percentage terms). 200 trials were presented with negative net money flow, half of which had positive ROI and the other half of which had negative ROI. All the trials were presented in a random order. Before formal experiment, participants practiced 30 trial runs.

**Figure 1:**
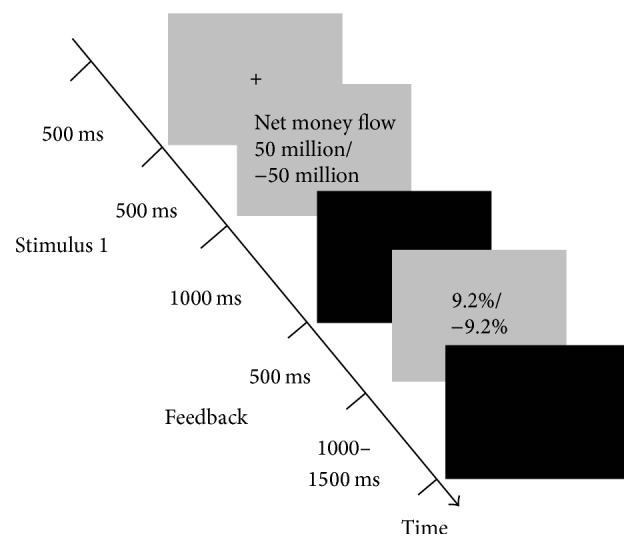
Experimental task.

At the beginning of each trial, a fixation “+” appeared as a cue for 500 ms on the screen, which was followed by the net money flow information for 500 ms. Participants were asked to make a decision, “to buy or not to buy,” within 1.5 s after the onset of net money flow information. Half of participants pressed right button for buying and pressed left for not buying, while the other half pressed the right button for not buying and left for buying. After the response, the ROI was displayed for 500 ms at the end of each trial. The interval between trials with a black screen lasted for 1000 ms to 1500 ms. The ERP paradigm could be seen in Figure 1. Participants would receive ¥ 30 as basic payment, in addition to which four trials would be randomly chosen to calculate their performance-based reward. If they made decision “to buy,” the ROI multiply by ¥ 30 to add into performance-based reward. If they made decision “not to buy,” no reward was added to performance-based reward. The final reward included basic payment and the performance-based reward. E-Prime 2.0 software package (Psychology Software Tools, Pittsburgh, PA, USA) was adopted to present stimuli, recording triggers and responses.

### 2.3. EEG Recording

EEG was recorded (band pass 0.05–100 Hz, sampling rate 500 Hz) with Neuroscan synamp2 Amplifier (Scan 4.3.1, Neurosoft Labs, Inc.), using Ag/AgCl electrodes placed at 64 scalp sites according to the extended international 10–20 system. The left mastoid served as online reference with a cephalic (forehead) location as ground. Vertical electrooculograms (EOG) were recorded with one pair of electrodes placed above and below the left eye, horizontal EOG with another pair 10 mm from the lateral canthi. Electrode impedance was maintained below 5 kΩ during the experiment.

### 2.4. Data Analysis

Before data analysis, the behaviors of “to buy” in positive net money flow condition and “not to buy” in negative net money flow condition can be classified into herding. The behaviors of “not to buy” in positive net money flow condition and “to buy” in negative net flow condition can be classified into antiherding. For the analysis of behavioral data, paired *t*-test was adopted to compare the choice rates and reaction times (RTs) across the herding and antiherding conditions.

For the analysis of EEG data, EOG artifacts were corrected using the method proposed by Semlitsch et al. [31]. Trials containing EOG activity or other artifacts (peak-to-peak deflection exceeding ± 80 *μ*V) were excluded from averaging. The remaining trials were digitally filtered with a low pass filter at 30 Hz (24 dB/Octave) and corrected to baseline. EEG recordings were extracted from −200 ms to 800 ms stimulus locked to the onset of ROI, with the period from −200 ms to 0 ms as baseline. For each participant, recorded EEGs were separately averaged over each recording site under each experimental condition. For the ROI, EEGs were separately averaged for behavior (herding/antiherding) × valence (positive/negative ROI) conditions. The valid trials for each subject under each condition exceeded 40, which could be proved by behavioral data.

Given that FRN arose in the anterior cingulate cortex [32], data from the electrode sites F3, Fz, F4, FC3, FCz, FC4, C3, Cz, and C4 were analyzed. Mean amplitudes in the 200–300 ms time window after onset of ROI, defined through visual inspection, went into 2 (behavior) × 2 (valence) × 9 (electrode) repeated measures ANOVA for FRN component. Considering that the maximum P300 amplitudes were observed at parietal sites, data of the electrode sites CP3, CPz, CP4, P3, Pz, P4, PO3, POz, and PO4 were analyzed. We averaged the ERP amplitude of the time window 300 ms to 450 ms after onset of the ROI. A 2 (behavior) × 2 (valence) × 9 (electrode) within-subjects repeated measure ANOVA for P300 was conducted. Simple effect analysis was conduct when the interaction effect was significant in the process of analyzing FRN and P300 components. The Greenhouse and Geisser correction was applied for the violation of sphericity assumption when necessary (uncorrected* df* were reported with the *ε* and corrected* p* values), and the Bonferroni correction was used for multiple paired comparisons.

## 3. Results

### 3.1. Behavioral Data

In the experiment, the percentage for choosing herding was 61.1% (s.d. = 0.02) and for choosing antiherding was 38.9% (s.d. = 0.02), which demonstrated a significant main effect [*t* (20) = 6.83, *p* < 0.001]. The average reaction time for herding was 451.336 ms (s.d. = 26.59 ms) and for antiherding behaviors was 453.121 ms (s.d. = 29.32 ms), which yielded no significant effect on RTs.

### 3.2. EEG Data

As the outcome evaluation was mainly reflected in the components of FRN and P300, all the following analyses referred to brain activity evoked by presentation of ROI information (stimulus locked). The ANOVA analysis for FRN revealed main effects of valence [*F* (1, 20) = 10.72, *p* < 0.01]. The main effect of valence showed more negative FRN responses to the negative ROI than to the positive ROI. However, the main effects of behavior and electrode were not significant. The valence factor interacted with behavior factor [*F* (1, 20) = 8.08, *p* < 0.05], suggesting that the FRN was modulated by herd or antiherd behavior. Further, the simple effect analysis was conducted and it was found that FRN amplitude was larger for the negative ROI than for the positive ROI in herding [*F* (1, 20) = 4.47, *p* < 0.05] and antiherding [*F* (1, 20) = 12.73, *p* < 0.01]. For the positive ROI, FRN evoked after herd behavior (M = 5.60, SE = 0.90) was more negative than that after antiherd behavior (M = 6.83, SE = 1.03) [*F* (1, 20) = 4.34, *p* ≤ 0.05]. However, for the negative ROI, there was no significant difference in FRN component between herding and antiherding.

**Figure 2:**
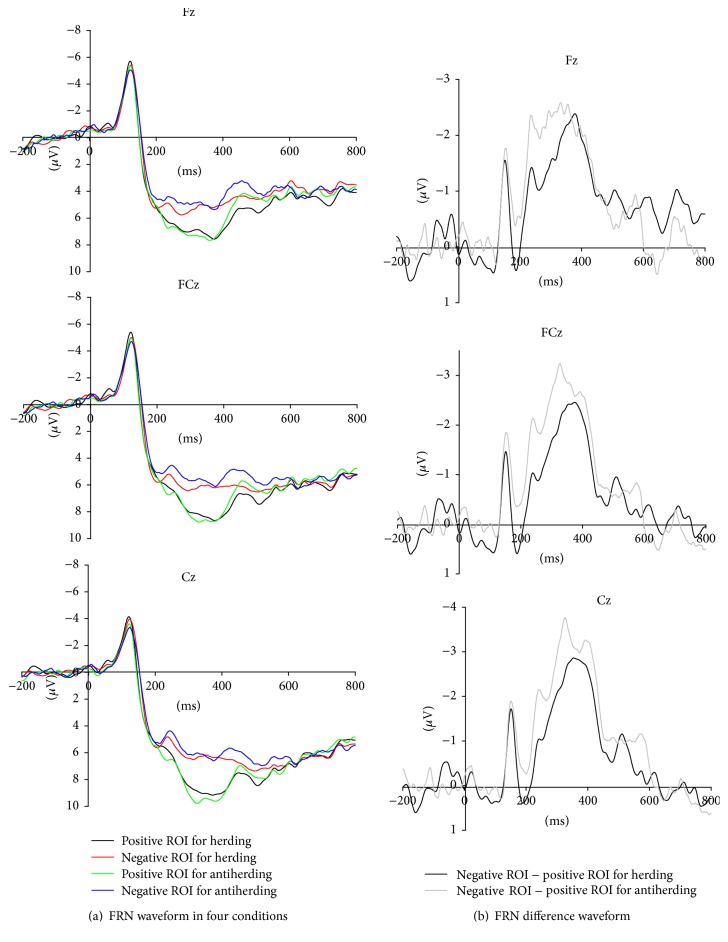
FRN results. The ERP Grand–average waveforms at channels Fz, FCz, and Cz in four conditions for FRN were presented in (a). Because the sensitivity to ROI was modulated by herding and antiherding, we further drew the difference waveforms for two conditions (negative minus positive ROI for herding and antiherding) in (b).

The significant interaction effects suggested that the sensitivity to ROI (negative ROI−positive ROI FRN) was affected by the behavior (herding or antiherding) of previous phase. The ANOVA analysis revealed a significant main effect on the differentiated FRN (d-FRN) (negative ROI minus positive ROI FRN) between herding and antiherding [*F* (1, 20) = 8.08, *p* ≤ 0.01], with a more negative d-FRN amplitude for antiherding condition (M = −1.87 *μ*V, SE = 0.53) than herding condition (M = −0.74 *μ*V, SE = 0.35). The FRN and d-FRN in herding and antiherding conditions were shown in Figure 2.

The ANOVA analysis for the P300 revealed main effects of valence [*F* (1, 20) = 32.40, *p* < 0.001] and electrode [*F* (8, 160) = 2.76, *ε* = 0.42, *p* < 0.05]. However, the main effect of behavior is not significant. The main effect of valence showed larger positive P300 responses to the positive ROI than to the negative ROI, which interacted with behavior factor [*F* (1, 20) = 4.93, *p* < 0.05]. We further analyzed the simple effect and found P300 was more positive for the positive ROI than for the negative ROI in herding [*F* (1, 20) = 29.39, *p* < 0.001] and antiherding [*F* (1, 20) = 26.81, *p* < 0.001]. For the positive ROI, the difference of P300 between herding and antiherding was marginally significant [*F* (1, 20) = 3.65, *p* < 0.1], and the amplitude of P300 elicited by antiherding (M = 9.46, SE = 1.25) was more positive than that by herding (M = 8.96, SE = 1.18). However, for the negative ROI, there was no significant difference in P300 component between herding and antiherding.

**Figure 3:**
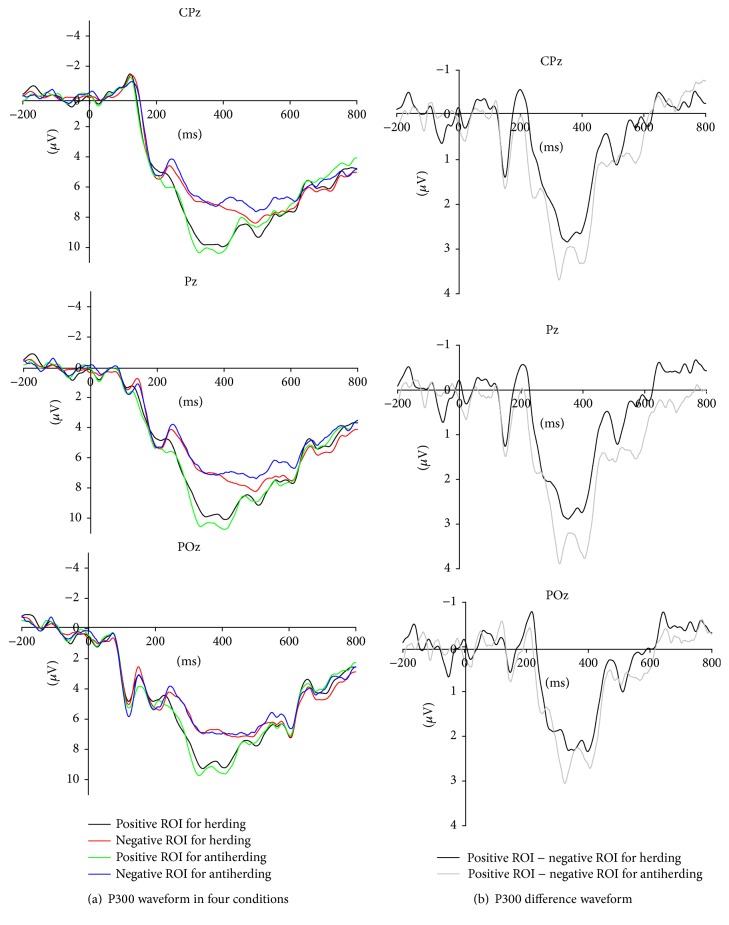
P300 results. The ERP Grand–average waveforms at channels CPz, Pz, and POz in four conditions for P300 were presented in (a). Moreover, we further drew the difference waveforms for two conditions (positive minus negative ROI for herding and antiherding) in (b).

The significant interaction effects suggested that the P300 was modulated by behavior types. The ANOVA analysis revealed a significant main effect on the differentiated P300 (d-P300) (P300 evoked by positive ROI minus P300 evoked by negative ROI) between herding and antiherding [*F* (1, 20) = 4.93, *p* < 0.05], with a more positive d-P300 amplitude for antiherding (M = 2.65 *μ*V, SE = 0.51) than herding (M = 1.84 *μ*V, SE = 0.34). The P300 and d-P300 in herding and antiherding were shown in Figure 3.

## 4. Discussion

In this study, we intended to investigate the temporal dynamics of how antiherding or herding affected the valuation of subsequent ROI under financial investment background. ERP results demonstrated that antiherding and herding indeed influenced the brain activity in the ROI evaluation, which could be reflected by FRN and P300 components. During the appearance of ROI, we observed larger FRN discrepancies (d-FRN) and P300 discrepancies (d-P300) towards positive ROI and negative ROI in antiherding condition than in herding condition. And, especially, in evaluating positive ROI process, the FRN and P300 evoked by antiherding were less negative and more positive than those evoked by herding, respectively. But in the evaluating positive ROI feedback, there was no significant difference for FRN and P300 component between herding and antiherding. Hence, the findings indicated that, compared with herding behavior, subjects who chose antiherding behavior were more sensitive to subsequent ROI, cared more about the outcomes of their choice, and put more attention on the outcome evaluation process, which could be reflected by d-FRN and d-P300. Moreover, the moderating effect of herding and antiherding on evaluating the ROI is mainly expressed in the process of valuing positive ROI.

Previous studies on herding often focused on how subjects made a judgment with the group opinion and controlled the different level of conflict evoked by changing the number of group members who made different choices from subjects' choice [8, 10, 12]. For example, Shestakova et al. (2013) studied how individual judgments of facial attractiveness were modulated by the group opinion [8]. In most of these studies, the choices of group influenced the subject's choice but with no relevance to subjects' outcomes (rewards) of decision-making and only affected the perception and cognition in the process of evaluating outcomes. But in financial market, the decision-making was not only affected by the group opinions of investors, but also affected by the capital amount invested by investors. In addition, the choices of group in investment decision-making had complex (direct or indirect) influences on the outcome of subject's choice. So in our study, one critical manipulation was the net money flow which could quantify the strength of buying group or selling group of a stock. Moreover, in the present study, the probability of positive ROI was at a 50% chance level; the dominated field choice (e.g., to buy in positive net money flow) was no better than alternative choice and provided no useful information to individuals. Thus, the results of our study minimized the learning effect from the feedback. In this experiment, we found some new findings about the herding and antiherding effect on the valuation of ROI at behavioral level and at the neural level.

From behavioral data, we found a larger percentage for choosing herding than antiherding. Previous research suggested that people were highly susceptible to social influence and had an intrinsic preference for conformity [2]. In financial market, investors could follow the actions of others to determine what was correct to do, or do what others do in order to “fit in” with the majority [2, 33], which could suggest that herding was more attractive for individuals than antiherding in our experiment. However, there was no significant difference in RTs between herding and antiherding. We speculated that subjects choosing antiherding behavior in investment decision-making believed their own choices would get better outcomes, while they choosing herding behavior thought group opinion was correct and would get better outcomes. In most cases, subjects trusted the opinion of group. Compared with herding behavior, there may be not some salient conflicts for subjects to choose antiherding, which resulted in no difference in RTs.

From ERP data, we found a general FRN effect for positive ROI and negative ROI, which was in accordance with previous findings that FRN could reflect the brain activity associated with different valences of the outcomes [15, 30]. We also found that behaviors of herding or antiherding modulated FRN effect. Antiherding evoked larger FRN discrepancies between positive ROI and negative ROI than herding. FRN amplitude was sensitive to outcome evaluation, and the different motivational/emotional significance could well explain the discrepancy in FRN electrophysiological responses [34, 35]. Previous studies have suggested that differences in FRN amplitude were correlated with subjects' subjective ratings of involvement and effort invested into the task [22, 34]. Li et al. (2010) suggested the responsibility leveled in performing a gambling task modulated the FRN effect, and the outcome revelation of FRN effect augmented to a greater extent in high responsibility condition [21]. In this study, the discrepancy in FRN (negative ROI–positive ROI) for antiherding behavior was more negative than that for herding behavior, which suggested herding or antiherding behavior would influence the motivational significance in the subsequent process of evaluating the ROI. For antiherding behavior, subjects were confident of their own information and ability to make correct choices and had intense motivation in the evaluating outcome process. For herding behavior, ROI as the feedback might have lost its motivational meaning for individuals.

From the results of simple effect analysis for FRN, herding and antiherding had a significant difference in evaluating positive ROI but with no difference in evaluating negative ROI. This finding demonstrated that herding and antiherding behaviors had greater modulation of the FRN to positive ROI than to negative ROI, which was consistent with the previous opinion that the effect of FRN was due to a positive deflection following positive outcomes [36–38]. In addition, this finding did not support the point that herding reduced the emotional impact of negative outcomes [32], because we found the differences for two kinds of behaviors in evaluating the negative outcomes were only visually observable but not statistically significant in our experiment. Previous research on the modulation to positive outcomes indicated that the more individuals were able to represent the correctness of the response, the less they relied on the feedback [38, 39]. Hence, the present findings were consistent with the idea that antiherding subjects were confident of the correctness of their own choices and reduced the positive outcome prediction error.

For the P300, the results also revealed a main effect in valence of ROI, which was in accordance with previous findings that the positive outcomes evoked larger P300 amplitudes than the negative outcomes in monetary gambling tasks [23]. Notably, different kinds of behaviors modulated the P300 difference waveform (positive ROI–negative ROI P300). Antiherding evoked larger amplitude of P300 difference waveform than herding. It was generally believed that P300 was implicated in a number of cognitive and affective processes and traditionally associated with allocation of mental resources [40]. In our study, if the decision maker took an action that goes against the herd, it suggested she/he not only was confident of his own choices but also sustained high stress. Hence, the attentional resources after antiherd behavior were paid more for the process of valuing the ROI, which led to a larger P300 discrepancy between positive ROI and negative ROI than herd behavior.

Moreover, from the results of simple effect analysis for P300 in parietal sites, herding and antiherding had a significant difference in evaluating positive ROI but with no difference in evaluating negative ROI. Previous studies indicated that the attention paid to the stimulus or event can modulate the effects of subjective motivational significance and probability on the amplitude of P300 [26, 27]. Thus, it suggested that two kinds of behaviors had an influence on the allocation of attention not paid to the negative ROI but to positive ROI, which modulated the amplitude of P300. Compared with herding, the mental resources after antiherding were paid more to the evaluation process of positive ROI (not negative ROI). This result further disconfirmed the opinion in social decision-making that herding protected individuals from experiencing negative emotions when the outcome was bad.

To sum up, we demonstrated a behavioral herding and antiherding effect when net money flow information was used in a simplified investment task. Applying the ERP technique, it was discovered that herding and antiherding shaped the valuation of outcomes (ROI), which was manifested on two points. On the one hand, the modulation effect of two kinds of behaviors could be reflected by the components of FRN and P300; on the other hand, herding and antiherding mainly had an effect on the evaluation process of positive outcomes, which proved that the effect of FRN was due to a positive deflection following positive outcomes. During the outcome stage, the discrepancies of FRN and P300 towards the positive and negative ROI were significantly enlarged in antiherding condition than in herding condition, which might suggest that, compared with herding, individuals under antiherding condition had stronger motivation and paid more attention in the evaluation process of ROI. Only for positive ROI, the amplitudes of FRN and P300 components were modulated by herding and antiherding, which suggested antiherding reduced the positive outcome prediction error and paid more mental resources to the positive outcomes. In general, such findings implied that individuals who made herding might be not confident in predicting the positive (not negative) outcomes and paid less attention to the evaluating positive (not negative) outcomes, which were inconsistent with the opinion that herding in social decision (social conformity) protected individuals from experiencing strong negative emotion when the outcomes were bad. This study may provide new insights into FRN and neurocognitive processes of herding and antiherding in financial market.
